# Evaluation of morphine, methadone, digoxin, and dronabinol poisoning during the years 2017 to 2019 in Ilam, Iran

**DOI:** 10.22088/cjim.14.2.356

**Published:** 2023

**Authors:** Kourosh Saki, Mahmoud Bahmani, Golnaz Zamanian, Naser Abbasi, Ali Aidy, Aliasghar Manouchehri, Sudip Kumar Mandal, Paramita Ganguly, Samira Shokri

**Affiliations:** 1 Department of Psychiatry, School of Medicine, Imam Hossein Hospital, Shahid Beheshti University of Medical Sciences, Tehran, Iran; 2 Biotechnology and Medicinal Plants Research Center, Ilam University of Medical Sciences, Ilam, Iran; 3 Experimental Medicine Research Center, Tehran University of Medical Sciences, Tehran, Iran; 4 Department of Pharmacology, School of Medicine, Tehran University of Medical Sciences, Tehran, Iran; 5 Department of Pharmacology, Ilam University of Medical Sciences, Ilam, Iran; 6 Department of Internal Medicine, Shahid Beheshti Hospital, Babol University of Medical Sciences, Babol, Iran; 7 Dr B.C. Roy College of Pharmacy and Allied Health Sciences, Meghnad Saha Sarani, Bidhan Nagar, Durgapur-713206, West Bengal, India; 8 Department of Pharmaceutical Technology, Brainware University, 398-Ramkrishnapur Road, Barasat, Kolkata-700125, West Bengal, India; 9 Department of Environmental Health Engineering, Division of Food Safety and Hygiene, School of Public Health, Tehran University of Medical Sciences, Tehran, Iran

**Keywords:** Poisoning drug, Dronabinol, Digoxin, Methadone, Morphine

## Abstract

**Background:**

Every year, drug poisoning is the most prevalent reason for referring patients to medical centers. This study aimed to evaluation of morphine, methadone, digoxin, and dronabinol poisoning in Shahid Mostafa Khomeini Hospital in Ilam.

**Methods:**

In this In this Cross-sectional study, patient samples suspected of morphine, methadone, digoxin, and dronabinol poisoning referred to the toxicology laboratory of Ilam University of Medical Sciences were analyzed using the HPLC method, and the results were analysed using SPSS software.

**Results:**

Results showed that the percentage of drug use is greater in men than in women. The highest percentage of morphine and methadone poisonings were detected in those under the age of 40, whereas the highest percentage of digoxin poisonings were recorded in those over the age of 80. As a result, the average age of digoxin users was substantially greater in men than in women. Methadone consumers showed significantly greater blood levels than others. In addition, there was a significant difference (P<0.01) in blood levels between men and women who used morphine.

**Conclusion:**

In general, it is important to understand the status of drug poisoning with drugs such as morphine, methadone, digoxin, and dronabinol, as well as the prognosis associated with the treatment process of such poisoning.

Every year, thousands of people die as a result of drug poisoning around the world. Poisoning occurs as a result of intentional or accidental exposure to potentially harmful medicines or chemicals (1, 2). Accidental poisoning is more common in children and individuals over the age of 50, but intentional poisoning, such as self-poisoning or suicide, is more common in adolescents and young adult (3, 4). In 2020, the age-adjusted rate of drug overdose deaths in the United States was 31% higher than the rate in 2019. In 2020, 91,799 drug overdose deaths occurred in the United States for an age-adjusted rate of 28.3 per 100,000 standard population (5). According to widely circulated figures in Iran, the fatality rate from poisoning is reported to be 8 per 1,000 hospitalized patients and 209 per 1,000 intensive care units (6). Poisoning is the most prevalent reason for hospitalization and the second highest cause of mortality among hospitalized patients in Iran (7). The factor of poisoning is different in various parts of Iran. Drug poisoning is widespread in Babol, Mashhad, and Tehran with sedatives and in northern Iran, notably in Gilan with pesticides and chemical fertilizers (8, 9). 

According to the statistics of the Forensic Medicine Organization in 2016, over 5,812 individuals died in Iran as a result of drug, toxins, and carbon monoxide poisoning, with drug poisoning having the greatest mortality rate (10, 11). Drugs that cause drug poisoning typically include amphetamines, opioids, methadone, heroin, hallucinogens such as LSD, antidepressants, dextromethorphan, pseudoephedrine, herbal stimulants, benzodiazepines, caffeinated pills, and energy drinks, as well as travel pills such as dimenhydrinate and diphenhydramine, heart medications, and so on (12-15). 

Methadone is a morphine-like pain reliever with a prolonged half-life of 25 to more than 52 hours. It alleviates withdrawal symptoms in those addicted to heroin or other narcotics. However, overdose might result in serious adverse effects or even die (16). Death from methadone poisoning is frequently caused by immune system suppression, while cardiac arrhythmias account for a large proportion of deaths. It involves nausea, lethargy, and drowsiness, and if appropriate treatment is not provided, the child may develop sleep apnea and even death (17, 18). 

Digoxin is a cardiac glycoside with a positive inotropic effect in the heart muscle that increases the intracellular concentration of sodium, decreases the cytoplasmic potassium, and finally increases the calcium required for the contractile proteins of the heart and increases strength by inhibiting the pump-ATPase Na+/K-.Myocardial contraction is used in patients with chronic congestive heart failure, atrial flutter, and acute atrial paroxysmal tachycardia (PAT) (19, 20).

Because the therapeutic and toxic doses of digoxin are very close to each other and have a narrow therapeutic index, small changes in serum digoxin levels can lead to severe intoxication; therefore, periodic evaluation of serum digoxin levels during treatment for prevention of poisoning is very important (21, 22).

 Dronabinol is a hallucinogenic isomer of tetrahydrocannabinol (THC), which is the major and most active isomer found in the Cannabis sativa L. plant, is also a pressing concern for global mental health (23). Dronabinol (Marinol), a synthetic cannabinoid, is used to treat anorexia in AIDS and other wasting diseases, emesis in cancer patients undergoing chemotherapy and chronic pain. Therapeutic doses of orally administered dronabinol range between 2.5 and 20 mg/day (24, 25).

According to a study in Iran (Mazandaran), the most common cause of poisoning leading to death was rice tablets and opium (26). Poisoning from opioids, tramadol, and pesticides (organophosphate and aluminium phosphide) has long been a concern in Iran. Also in many countries, various studies have been done about drug types and poisons along with their symptoms, complications, and mortality rates in high risk and deceased patients. 

In comparison with other countries, drugs are easily accessible in Iran. Few studies have investigated the medicinal poisoning pattern in our country. There is a necessity to know the medications that contribute to poisoning cases, to take up appropriate preventive efforts and management. So according to the importance of the issue and the prevalence of drug poisoning leading to death, this study aimed to this study aimed to evaluation of morphine, methadone, digoxin, and dronabinol poisoning in Shahid Mostafa Khomeini Hospital in Ilam, Iran as well as the prognosis associated with the treatment process of such poisoning.

## Methods

The current research is a cross-sectional study. The present study with IR code of ethics. MEDILAM.REC.1401.148 was conducted at Ilam University of Medical Sciences. The study population included all the poisoned people referred to Shahid Mostafa emergency hospital during 2017 to 2019 years. Shaheed Mostafa Hospital in Ilam city is the toxicology and clinical poisoning center in Ilam province. Between the years 2017 and 2019, blood and serum samples of suspected patients who were referred to the hospital laboratory were coded and finally the samples were tested for morphine, methadone, digoxin and dronabinol drugs. Based on the method of Schulz and Schmoldt, 2003, a serum level higher than the standard was considered as poisoning (27).

 In this study, samples suspected of poisoning with morphine, methadone, digoxin and dronabinol drugs referred to the toxicology laboratory of Ilam University of Medical Sciences were measured by HPLC method. In fact, the patients of this study were people who had neurological and digestive symptoms caused by drug overdose and were referred to the poisoning department of the hospital. Also written consent was obtained from the patients participating in the study and assurance that their personal information will remain confidential. Results are reported based on µg/mL. Since these four drugs have many medicinal uses and the possibility of poisoning with them is high, they were chosen. Above the standard level of serum level of any drug in the blood, the criterion of blood poisoning was considered. 

**Morphine assay: **The UV spectrum of morphine was first established using a Photodiode Array Detector to determine the maximum wavelength for ultraviolet detection. The column temperature was maintained at 25ºC and the detection wavelength was set at 226 nm. The total run time was set at 7 minutes with a flow rate of 1 ml/min and the injection volume was 20 µl.

 The separation was carried out using the mobile phase (pH 7.0) consisting of acetonitrile: bi-distilled water: methanol (60: 840: 100, v/v/v) with the addition of 0.012 M monopotassium phosphate, and 0.5 mM ethylenediaminetetraacetic acid disodium salt. Data collection and processing were carried out using Chromgate software (20). A total of 203 samples were tested for the presence of morphine.

**Methadone assay: **The RP-HPLC with diode array detection (RP-HPLC–DAD) system was used to develop a method for the simultaneous determination of methadone in plasma. Briefly, the separation was carried out using the gradient elution of acetonitrile and 0.02 M phosphate buffer pH 6.5 under a detection wavelength of 292 nm. 

The injected volume was 20 ml and the run time was 22 minutes (21). A total of 104 samples were tested for the presence of methadone.

**Tetrahydrocannabinol assay: **THC was extracted from plasma samples using protein precipitation with cold acetonitrile and liquid-liquid extraction with n-hexane. Following centrifugation, the upper organic layer was recovered and dried before being reconstituted in acetonitrile. An aliquot of 30 µl was injected into the HPLC column. Separation was achieved using an ACE C18-PFP, 150 x 4.6 mm, 3 µm column with isocratic elution of acetonitrile-water (62: 38, v/v). 

The column temperature was set to 55 °C with a flow rate of 1 ml/min and a run time of 20 minutes. Both cannabis and the reference standard (4, 4-dichlorodiphenyltrichloroethane, DDT) were identified at 220 nm (22). A total of 41 tetrahydrocannabinol samples were tested for the presence of dronabinol.

**Digoxin assay: **The procedure established in the previous study was used to extract digoxin from plasma samples (23). In brief, the plasma sample was extracted using chloroform-isopropanol (9: l) after a pre-wash with isooctane to eliminate endogenous compounds in order to recover maximum digoxin (70%). Using digitoxigenin as the internal standard, the calibration curve was linear (r = 0.9999) across the range of 0.5–4 ng/ml of digoxin in plasma (23). The presence of digoxin was determined in 32 samples.

**Instrumentation and chromatographic analysis: **Knauer’s PLATINblueRP-HPLC system (Knauer company, Germany) was used to analyze the samples.The separation was carried out using Eurospher II 100-5 C18,250 × 4.6 mm column.

**Statistical analysis: **The Mann-Whitney test was used to analyze the data using SPSS statistical software. The columns were categorized according to the IQR Median.The data were presented as mean ± SD, with a p value less than 0.05 considered significant.

## Results

**Figure 1:**
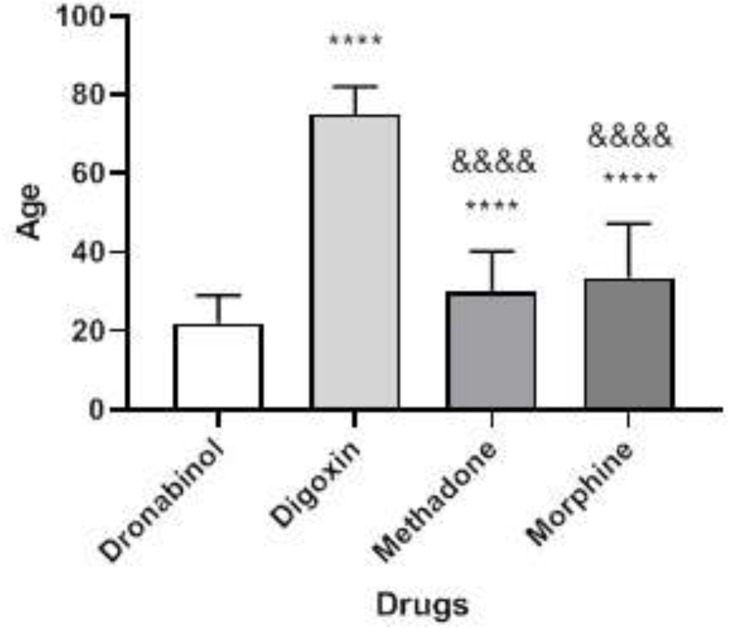
Relationship between age and drug use in patients. The ‘*’ mark was compared to the dronabinol group and the ‘&’ mark was compared to the digoxin group. Mark “****” or “&&&&” = P<0.0001

**Figure 2:**
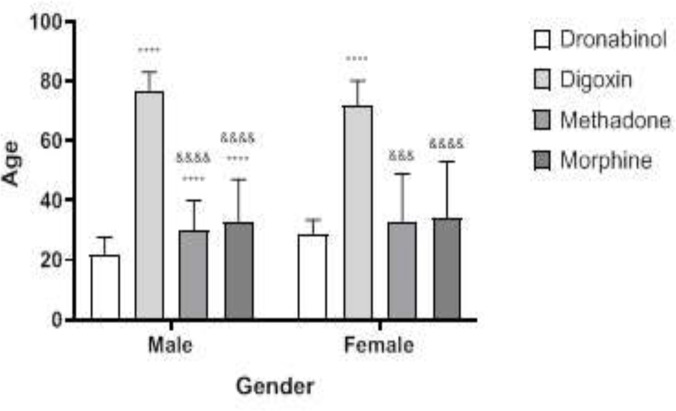
Relationship between age, sex and drug use in patients. The ‘*’ mark was compared to the dronabinol group and the ‘&’ mark was compared to the digoxin group. Mark **** or &&&& = P<0.0001. The average age of digoxin users was significantly higher in men than in women

**Figure 3:**
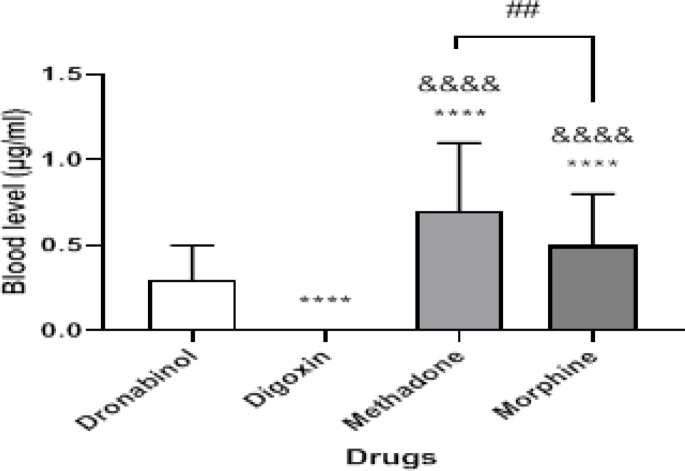
Relationship between blood level and drug use in patients. The ‘*’ mark was compared to the dronabinol group and the ‘&’ mark was compared to the digoxin group. Mark **** or &&&& = P<0.0001 and ## = P< 0.01

**Figure 4:**
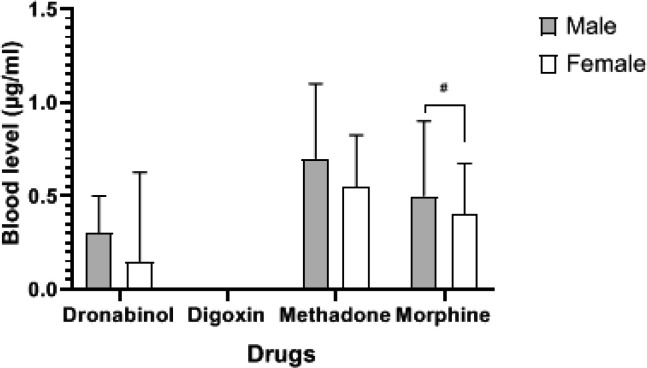
Relationship between blood level, sex, and drug use in patients. Mark # = P<0.05

The highest percentage of morphine and methadone poisonings occurred in adults under to the age of less than 40 years, whereas the highest percentages of digoxin poisonings were in those over the age of 80 years. The age of morphine and methadone intake varied from that of digoxin and dronabinol (P < 0.0001) (figure 1). As a result, the average age of digoxin users was substantially greater in men than in women (figure 2). There were significant differences in plasma concentrations between morphine and methadone users. Methadone users showed higher blood levels than others (P < 0.01) (figure 3). There was a significant difference in plasma concentrations between men and women who used morphine (P < 0.05) (figure 4). The percentage of drug use is greater in men than in women (table 1).

**Table 1:** Percentage of men and women in the use of drugs

**Sex**	**Dronabinol**	**Digoxin**	**Methadone**	**Morphine**
**Male%**	80.49	53.13	89.58	81.57
**Female%**	19.51	46.88	10.42	18.43

## Discussion

Excessive drug consumption is always associated with the risk of serious adverse effects, including overdose (27). The current study, like most others in Iran, found it to be the most common cause of drug poisoning in Urmia (28), Shiraz (29), Isfahan (30), Tabriz (31), Sari (4), and other parts of the world, including China (32), and Oslo (33), it was the most common cause of drug poisoning. Excessive prescription of drugs by doctors, easy access, and over-the-counter drug sales may be contributing factors to the increased prevalence of drug poisoning (29). As well asThe existence of underlying issues such as social, cultural, economic, psychological, and fluency problems, and especially drug addiction in any society can lead to intentional (34). Results of the current study showed that the majority of use drugs were male, which is consistent with the findings of Aryaie and Burillo studies (35-37). However, in Fazlohah's studies, the ratio of women to males was greater, which is contradictory (25). According to statistics, men had the biggest number of accidental poisonings, while women had the highest number of suicide attempts (38).

In various age groups, the type of morphine and methadone drug intoxication with digoxin and dronabinol was significantly different. Figure 1 depicts the highest and lowest age ranges for digoxin and dronabinol poisoning. Aging is a risk factor for digoxin toxicity. In this context, Baharvand (39) established a correlation between serum digoxin levels and patient age. Patients over the age of 65 years clearly showed signs of clinical intoxication at lower concentrations of digoxin in the serum (40).The therapeutic and toxic levels of digoxin in the serum vary from person to person, and the gap between serum digoxin levels and toxin levels is relatively narrow (41). 

However, its serum level of drugs is affected by a variety of factors, including renal function, hepatic excretion (to a lesser extent), and the influence of other medicines. In 62% of patients, renal function enzymes were elevated. The most important risk factor for digoxin intoxication was an exacerbation of preexisting renal diseases or acute renal failure (42). Therefore, digoxin should be administered with caution on a daily basis. Several studies have shown a correlation between high levels of digoxin poisoning and mortality (43).

 Methadone, a synthetic and long-acting opioid, is the second most common cause of poisoning. Unfortunately, consumption, emergency visits, and associated deaths have increased globally in recent years. The same dose of methadone might have distinct effects on different people. This medication has a lengthy duration of action and is both an analgesic and a narcotic (44). The analgesic effect of this drug is short-lived, lasts about 4-6 hours, and its elimination half-life is between 24-48 hours (45). In the current study, there was a significant difference in serum concentrations between morphine and methadone use. Methadone users had greater serum concentrations than other drug users. This medication has a very strong first-pass effect on the liver. Methadone is transported in the blood and tissues by binding to albumin and other proteins in the lungs, kidneys, liver, and spleen. Methadone causes death through a variety of mechanisms, including respiratory depression, aspiration pneumonia, pulmonary edoema, and heart problems (46).

Methadone has played a significant role in child poisoning due to the increased occurrence of accidental poisoning in children. In a study that compared the characteristics of methadone poisoning due to methadone pills and syrup in 1426 cases of methadone poisoning in country poisoning centres from 2000 to 2010 AD, the most accidental methadone poisoning occurred in children under the age of 12. The cause of misuse instead of breast syrup or water has been reported. In contrast, the majority of the poisonings were caused by methadone pills combined with suicidal intent (27).

Morphine is rapidly absorbed from the GI tract, has a bioavailability of around 43%, and is significantly affected by first-pass hepatic metabolism. It spreads throughout the body, with the liver, kidneys, lungs, and spleen being the most affected. More than 13% of a dose is conjugated in the faeces, with the balance conjugated in the urine (47). The principal complication of morphine is respiratory failure caused by direct CNS depression, which can eventually progress to apnea or total respiratory arrest (48, 49).

The finding showed that the quantity of morphine in the blood and muscles ranged from 0.2 to 2.8 micrograms per gram in 14 cases of fatal poisoning, 10 of which were caused by intravenous morphine (50) According to the current study, dronabinol poisoning is more common among adolescents. According to Bridget Onders, from 2000 to 2013, there were 1969 marijuana exposures among children aged 6 years old, with an exposure rate of 5.90 per million children (51)

In the Vanani study, the highest frequency of abusers was associated with 30 years of age, and the male/female ratio was 4 to 1. Methamphetamine had the highest rate of use among the drugs tested. In this study, 55.7% of the patients required admission to the critical care unit, and two deaths occurred. The most common symptom among poisoned patients is loss of consciousness, whereas respiratory distress is the least common (52).

Although chemical and herbal medicines can be beneficial in the treatment of diseases and disorders (53-55), such drugs have side effects, are toxic in high doses, and cause poisoning (49-54). In recent years, drug poisoning, especially drugs such as morphine, methadone, digoxin, and dronabinol, has become common in Ilam province. Therefore, we decided to study drug poisoning in a seroepidemiological way to prevent serious complications and treat them by timely diagnosis of such poisonings. The extent of opioid analgesics use varies widely in the Iran. Variation in the availability of opioid analgesics is related to the spatial distribution of drug poisoning mortality. So, epidemiological information in a regional form leads to the rational use of resources for the prevention and control of poisoning. Also early prevention, as well as informing and educating people, can be very helpful.
